# Silent Trichophagia Behind Visible Trichotillomania: A Case Report of a Giant Gastric Trichobezoar in an Adolescent With Intellectual Disability and Anemophobia

**DOI:** 10.7759/cureus.112285

**Published:** 2026-07-08

**Authors:** Fatiha Belaziz, Houda Koreda, Fatima Benkarroum, Jihane Moussaoui, Mohamed Barrimi

**Affiliations:** 1 Psychiatry, Centre Hospitalo-Universitaire Mohammed VI d'Oujda, Oujda, MAR; 2 Psychiatry, Mohammed VI University Hospital, Oujda, MAR; 3 Psychiatry, Faculty of Medicine and Pharmacy of Oujda, Mohammed First University, Oujda, MAR; 4 Immunohematology Cellular Therapy, Faculty of Medicine and Pharmacy of Oujda, Mohammed First University, Oujda, MAR; 5 Immunohematology Cellular Therapy, Mohammed VI University Hospital Center, Oujda, MAR

**Keywords:** adolescent, anemophobia, case report, intellectual disability, trichobezoar, trichophagia, trichotillomania

## Abstract

Somatic complications of trichotillomania arising from undetected trichophagia are not well characterized in adolescents with intellectual disability. We report a case of a 16-year-old girl with intellectual disability whose clinical picture unfolded in four successive steps over four years: untreated anemophobia for two years despite an outpatient consultation, trichotillomania emerging two years later, silent trichophagia concealed from the family for one year, and finally a giant gastric trichobezoar revealed by hair-containing vomiting, associated with severe iron-deficiency anemia. Following combined management, including surgery, sertraline, and family psychoeducation, trichophagia resolved completely, while residual trichotillomania episodes without hair ingestion persisted at outpatient follow-up. This case highlights the need for systematic and active screening for trichophagia in any patient followed for trichotillomania, even when hair-pulling is visible and known to the family, and underscores the importance of identifying and managing comorbid anxiety disorders in the management of this condition.

## Introduction

Trichotillomania (TTM), classified among the obsessive-compulsive and related disorders (OCRDs) in the Diagnostic and Statistical Manual of Mental Disorders, Fifth Edition, Text Revision (DSM-5-TR) [1], is characterized by recurrent pulling out of one’s own hair, resulting in clinically visible hair loss. Its prevalence is estimated at 1.7% in the adult general population, with a mean age of onset of 14.8 years in females [2]. Psychiatric comorbidity is common: in a Swedish national cohort of 1,234 patients, 79% had at least one associated psychiatric disorder, including anxiety disorders in 65% and neurodevelopmental disorders in 39% [3].

More than 20% of patients with TTM ingest the hair they pull, a behavior known as trichophagia [4]. This behavior is often concealed by the patient and may go unrecognized by the family [5]. When chronic, ingested hair accumulates in the gastric cavity to form a trichobezoar, a compact mass that resists digestion due to its keratin composition, and may cause obstruction, ulceration, or digestive hemorrhage [6-8].

Anemophobia refers to an intense, persistent fear of wind and of objects set in motion by wind. It is not a distinct entity in the DSM-5-TR but corresponds to a specific phobia of the natural-environment type [1]. We report this case in light of the distinctive temporal sequence of the disorders and their occurrence in a patient presenting with both intellectual disability and anemophobia, a combination that has not, to our knowledge, been previously described in this specific configuration.

## Case presentation

The patient was a 16-year-old girl, the younger of two siblings. She was referred to the pediatric emergency department by a private-practice pediatrician for recurrent abdominal pain and vomiting containing hair. Personal and family history, both somatic and psychiatric, was unremarkable. There was no history of psychological trauma or substance use. The patient had no friendships outside the family, and her mother described her as notably less socially interactive than her sister.

The patient had an intellectual disability, retained on clinical and developmental grounds in the absence of formal psychometric assessment, which was not performed owing to limited resources. Developmental milestones were delayed, with first words and walking achieved around 24 months, and schooling was discontinued after fifth grade. She struggled with basic calculations and could not correctly set a clock to a given time; had no friendships outside the family; and required supervision for bathing and could not go out unaccompanied.

History-taking allowed reconstruction of a precise four-year sequence. Around age 12, the patient developed an intense fear of wind and of any object that could be set in motion by it (leaves, branches, curtains, or clothing), making outdoor exposure on windy days unbearable and progressively leading to school dropout. This fear prompted a single outpatient consultation around that age, but no diagnosis was made, and no treatment was initiated. Two years later, around age 14, compulsive hair-pulling appeared and was dismissed by the family as a simple nervous habit. The patient avoided discussing where she pulled from or how often. One year after that, around age 15, the patient began ingesting the pulled hair, which was unknown until the current presentation.

Approximately one month before admission, the patient experienced intermittent abdominal pain of moderate intensity, associated with alternating episodes of diarrhea and constipation, decreased appetite, and weight loss that set in gradually. In the week before the emergency visit, two episodes of hair-containing vomiting appeared, which prompted the family to seek medical attention for the first time.

On admission, vital signs were as follows: blood pressure of 120/70 mmHg, heart rate of 93/min, respiratory rate of 21/min, temperature of 37°C, and oxygen saturation (SpO₂) of 98%. Weight was 37 kg, height was 152 cm, and BMI was 16.0 kg/m².

On physical examination, the patient had frank cutaneous-mucosal pallor, epigastric tenderness on palpation, and a palpable abdominal mass on deep pressure. Organic and toxic causes were systematically ruled out based on a full blood panel, ECG, and abdomino-pelvic CT scan, which showed no abnormality other than the bezoar. Laboratory findings revealed severe iron-deficiency anemia, with a hemoglobin level of 7.5 g/dL, white blood cell count of 4,800/μL, and ferritin < 1 ng/mL, as presented in Table 1.

**Table 1:** The results of the patient's biological assessments. MCV: mean corpuscular volume; MCH: mean corpuscular hemoglobin; MCHC: mean corpuscular hemoglobin concentration; CRP: C-reactive protein; ASAT: aspartate aminotransferase; ALAT: alanine aminotransferase.

Parameter	Patient's value	Reference range
Red blood cells	4.59 × 10⁶/μL	3.8–5.4 × 10⁶/μL
Hematocrit	28.4%	37–47%
MCV	61.9 fL	82–98 fL
MCH	16.3 pg	27–33 pg
MCHC	26.4 g/dL	32–36 g/dL
Platelets	316 000/μL	15000–400000 /μL
White blood cells	4800/μL	4000–10000/μL
CRP	3.32 mg/L	0–10 mg/L
Ferritin	<1.00 ng/mL	4.63–204 ng/mL
Sodium	136 mEq/L	136–145 mEq/L
Potassium	4.0 mEq/L	3.5–5.1 mEq/L
Chloride	107 mEq/L	98–107 mEq/L
Bicarbonate	21 mmol/L	21–28 mmol/L
Urea	0.15 g/L	0.15–0.45 g/L
Creatinine	5.08 mg/L	5.7–11.1 mg/L
ASAT	28 IU/L	5–34 IU/L
ALAT	21 IU/L	0–55 IU/L
Total protein	71 g/L	60–80 g/L
Albumin	42 g/L	35–50 g/L
Calcium	90 mg/L	84–102 mg/L
Triglycerides	0.58 g/L	<1.50 g/L
Total cholesterol	1.10 g/L	<1.70 g/L
Vitamin B12	540 pg/mL	187–883 pg/mL
Folic acid	3.80 ng/mL	2.34–17.56 ng/mL

**Figure 1:**
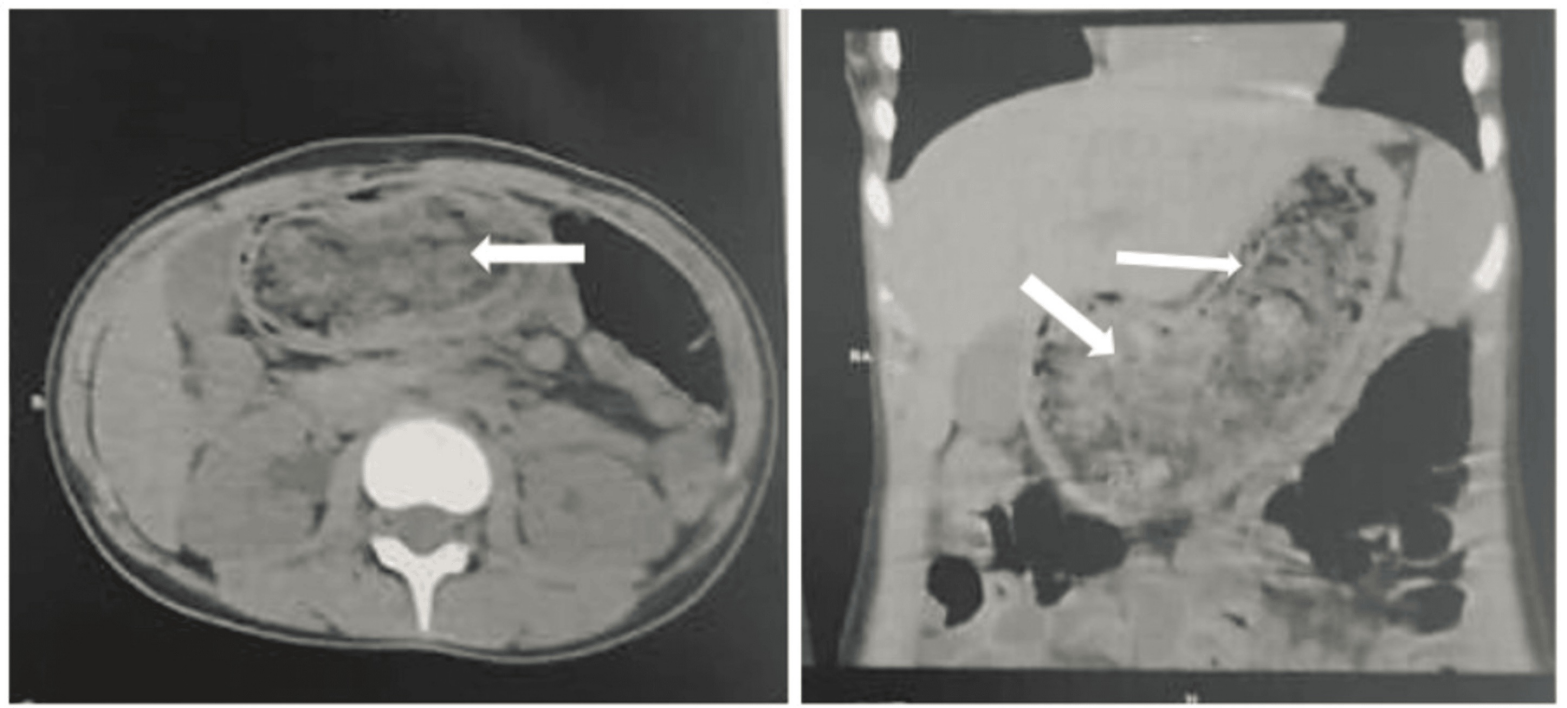
Contrast-enhanced CT scan (axial and coronal views) showing a large heterogeneous intragastric mass consistent with a giant trichobezoar, without intestinal extension.

Abdominal ultrasound showed a hyperechoic intragastric image with posterior acoustic shadowing consistent with an organized foreign body. Contrast-enhanced abdomino-pelvic CT confirmed a distended stomach containing a large, heterogeneous, thick-walled mass without intra-intestinal extension, as shown in Figure 1.

Surgical treatment consisted of open gastrotomy with en bloc extraction. Dimensions and weight of the bezoar were not recorded. Progressive clinical improvement was noted from approximately the 10th postoperative day. The hospital stay was approximately three weeks. The patient received oral ferrous sulfate, 80 mg, two tablets daily. A complete blood count performed at three months showed hemoglobin at 8 g/dL, and iron supplementation was continued accordingly.

Postoperative psychiatric assessment found depressed mood, impoverished thinking, and a tendency to minimize symptoms. No psychotic signs were identified. The retained diagnosis was trichotillomania with trichophagia, combined with anemophobia (specific phobia, natural-environment type) and intellectual disability. Sertraline was started at 25 mg/day, increased to 50 mg/day after one month, then to 75 mg/day after three months, and maintained at that dose for one year, alongside family psychoeducation. At follow-up, trichophagia resolved completely under combined management; residual TTM episodes without ingestion persisted, reported as rare by the mother despite close supervision.

The main clinical, paraclinical, and therapeutic features of this case are summarized in Table 2.

**Table 2:** Clinical timeline from onset of psychiatric symptoms to outpatient follow-up. TTM: trichotillomania.

Time period	Clinical event
Around age 12	Onset of intense fear of wind and any wind-moved object (leaves, branches, clothing, curtains). A single outpatient consultation without a diagnosis or treatment. Progressive school dropout.
Around age 14	Onset of compulsive hair-pulling (trichotillomania). Dismissed by family as a nervous habit.
Around age 15	Silent onset of trichophagia (ingestion of pulled hair). Behavior concealed from family, unknown until the current presentation.
One month before admission	Recurrent abdominal pain, alternating diarrhea and constipation, progressive pallor, and weight loss.
Day of admission (D0)	Hair-containing vomiting revealed trichophagia to the family for the first time. First medical consultation in the emergency department. Severe iron-deficiency anemia. Abdominal ultrasound then CT scan: a giant gastric trichobezoar without intra-intestinal extension.
Surgical admission	Open gastrotomy with en bloc extraction of the trichobezoar. Simple postoperative course. Progressive correction of anemia with oral iron supplementation.
Postoperative psychiatric assessment	Depressive mood, impoverished thinking, minimization of behaviors. Diagnosis: TTM with trichophagia, anemophobia, and intellectual disability. Sertraline initiated at progressive doses + intensive family psychoeducation.
Outpatient follow-up	Complete resolution of trichophagia. Persistence of rare residual TTM episodes without ingestion. Outpatient psychiatric follow-up was maintained over a total period of one year.

## Discussion

Rationale for the diagnosis

TTM with trichophagia was supported by patient-acknowledged hair-pulling, hair-containing vomiting, and an intraoperative trichobezoar [4,5]. Anemophobia met DSM-5-TR criteria for a specific phobia given the years-long, situation-specific fear and avoidance [1]. Intellectual disability was based on developmental delay (first words and independent walking around 24 months) and adaptive deficits spanning three domains: conceptual (difficulty with calculation and inability to correctly set the hands of a clock to a given time), social (limited rapport and brief and poorly elaborated responses), and practical (need for supervision with bathing and inability to manage a simple purchase or go out independently), in the absence of formal intelligence quotient (IQ) testing. Organic causes were excluded by laboratory work, ECG, and CT.

Anxiety comorbidity in trichotillomania

Anemophobia preceded TTM by two years, consistent with the well-documented overlap between TTM and anxiety disorders, present in 65% of TTM patients [3]. The consultation had led to neither a diagnosis nor treatment. Exposure-based cognitive-behavioral therapy, typically delivered as a single, massed session of graded exposure, is the established first-line approach for specific phobias in children and adolescents aged seven to 17 years [9]. Wind phobia specifically has been documented in a pediatric case successfully treated with cognitive-behavioral therapy combined with a low-dose selective serotonin reuptake inhibitor (SSRI) [10]. Its main precaution is pacing exposure to avoid dropout, and its principal limitation is the patient's capacity to actively engage with the protocol, which was constrained in our patient by her intellectual disability. Pharmacotherapy with an SSRI is a second-line, adjunctive option when anxiety persists despite psychological intervention; sertraline, the SSRI used in this case, is discussed below with its dosing, monitoring, and adverse-effect profile. Addressing comorbid anxiety is a relevant component of comprehensive TTM management [3,11,12].

Undetected trichophagia

For a full year, the family had witnessed hair-pulling without ever suspecting ingestion. This gap between the visibility of TTM and the invisibility of trichophagia is well documented: Klobučar et al. note that patients frequently deny hair ingestion and actively conceal it, even when pulling is known and observed by those around them [5]. The same pattern appears across several recent case series [6-8] and carries a direct clinical implication: visible TTM offers no protection against bezoar formation.

Hair-containing vomiting is a revealing sign of a trichobezoar [6,7]. The iron-deficiency anemia observed was likely multifactorial, with decreased appetite and mucosal erosion from chronic contact both described as contributing mechanisms in similar cases [7]. Active and repeated screening for trichophagia should be built into the routine follow-up of every patient with TTM, regardless of how mild the hair-pulling appears [5-8].

Intellectual disability as a complicating factor

Diagnostic Impact

Cooper et al. (2007) found 40.9% of adults with intellectual disabilities had a significant psychiatric disorder, with low specialist consultation rates [11]. Cognitive limitations here reduced the patient’s ability to recognize and report her behaviors.

Therapeutic Impact

Habit reversal training (HRT) is a structured behavioral intervention whose core components are awareness training (recognizing the behavior and its triggers), competing response training (substituting an incompatible motor action when the urge to pull arises), and stimulus control (modifying the environmental situations associated with hair-pulling) [13]. Acceptance and commitment therapy has also shown efficacy as a standalone treatment for TTM in both adults and adolescents [14]. The meta-analysis by Bloch et al. (2007) demonstrated that HRT outperformed pharmacotherapy in TTM, with significant efficacy against placebo that SSRIs alone did not achieve [12]. Combining both approaches produces better outcomes than either alone [15]. No distinct psychotherapeutic protocol exists specifically for trichophagia; ingestion is managed within the same HRT framework as hair-pulling [16]. In our patient, HRT could not be implemented owing to her cognitive limitations and the limited local availability of adapted protocols.

Sertraline was selected for its established efficacy in OCRDs, the category under which TTM is classified in the DSM-5-TR [1]. It is approved for obsessive-compulsive disorder (OCD) in children and adolescents aged six to 17 years, with a maximum dose of 200 mg/day [12,15]. Close monitoring for suicidal ideation is recommended in adolescents, particularly during the first weeks of treatment and at dose changes [12]. Common adverse effects include nausea, insomnia, and headache; abrupt discontinuation can produce a withdrawal syndrome, so gradual tapering is recommended when treatment is stopped.

Surgically, large impacted bezoars such as this one require open or laparoscopic gastrotomy rather than endoscopic removal, which carries a significant failure rate and risk of esophageal injury [17]. Outcomes are consistently good across age groups, adolescents included [17]. As with any open abdominal procedure, the main risks are bleeding, surgical site infection, and postoperative ileus [17].

Trichophagia resolved following concurrent surgical extraction, sertraline, and family psychoeducation; as these interventions were applied simultaneously, their individual contributions cannot be determined.

Limitations and strengths

Several limitations should be noted. No standardized TTM severity scale was used. A formal neurological examination and formal psychometric assessment were not performed. Bezoar dimensions were not recorded.

Despite these limitations, this report offers a detailed reconstruction of a four-year clinical sequence and documents the constraints of resource-limited settings on care (including the unavailability of formal psychometric testing and HRT), information relevant to clinicians practicing in comparable contexts. Future research should examine the prevalence of undisclosed trichophagia among TTM patients and develop HRT protocols adapted for patients with intellectual disability.

## Conclusions

When trichophagia is not actively sought, visible trichotillomania may be mistaken for a benign condition. Addressing comorbid anxiety is a relevant component of TTM management. Trichophagia resolving under combined surgical, pharmacological, and psychoeducational management supports a multidisciplinary approach. Active screening for trichophagia should be integrated as a standing routine in every follow-up visit for any patient with trichotillomania.
